# Virus-Like Particles as an Immunogenic Platform for Cancer Vaccines

**DOI:** 10.3390/v12050488

**Published:** 2020-04-27

**Authors:** Jerri C. Caldeira, Michael Perrine, Federica Pericle, Federica Cavallo

**Affiliations:** 1 AgilVax, Inc., Albuquerque, NM 87110, USA; jcaldeira@agilvax.com (J.C.C.); mperrine@agilvax.com (M.P.); 2 Border Biomedical Research Center, University of Texas at El Paso, El Paso, TX 79968, USA; federica_pericle@outlook.com; 3 Department of Molecular Biotechnology and Health Sciences, Molecular Biotechnology Center, University of Torino, 10124 Torino, Italy

**Keywords:** virus-like particles, vaccine, cancer, immunotherapy

## Abstract

Virus-like particles (VLP) spontaneously assemble from viral structural proteins. They are naturally biocompatible and non-infectious. VLP can serve as a platform for many potential vaccine epitopes, display them in a dense repeating array, and elicit antibodies against non-immunogenic substances, including tumor-associated self-antigens. Genetic or chemical conjugation facilitates the multivalent display of a homologous or heterologous epitope. Most VLP range in diameter from 25 to 100 nm and, in most cases, drain freely into the lymphatic vessels and induce antibodies with high titers and affinity without the need for additional adjuvants. VLP administration can be performed using different strategies, regimens, and doses to improve the immunogenicity of the antigen they expose on their surface. This article summarizes the features of VLP and presents them as a relevant platform technology to address not only infectious diseases but also chronic diseases and cancer.

## 1. Introduction

Vaccines are among humanity’s most significant achievements. By the end of the 20^th^ century, our efforts to prevent infectious diseases through vaccination and public sanitation were so successful that life expectancies had increased by approximately 30 years. Historically, vaccines have been used primarily as measures to prevent infectious diseases, but that focus has begun to shift as new strategies emerged on their possible use as treatments for certain chronic infectious diseases and cancer. The challenge is to find technical approaches that maximize immunogenicity without compromising safety, tolerability, and efficacy. Virus-like particles (VLP) are an attractive option.

Over the last three decades, genetically or chemically modified VLP have been used to present different classes of epitopes on their surface for diverse applications. Their ability to induce humoral and cellular response has been confirmed by many preclinical and clinical studies [1] and has offered a substantial advancement in the vaccine field. VLP vaccines are used to induce immune response against several diseases in animals [2] and humans. Some examples of treatment are osteoporosis [3], chronic pain [4], type 2 diabetes [5], and tauopathy [6]. Further, other studies have addressed the possibility of using VLP to stimulate the immune response against cancer antigens.

Here, we describe the main features of VLP as a vaccination strategy and their interaction with the immune system before focusing on VLP as cancer vaccines.

## 2. VLP as a Vaccination Strategy

VLP are nanoparticles that are spontaneously assembled from viral structural proteins. Structurally, they are practically indistinguishable from their corresponding viruses and, a VLP derived from the structural proteins of a viral pathogen can often serve as a highly effective vaccine for that pathogen. The structural protein of Human Papillomavirus (HPV), for example, assembles into a VLP that elicits antibodies that protect against HPV infection and thereby prevent cervical cancer. Similarly, the Hepatitis B Virus (HBV) surface antigen self-assembles into a VLP vaccine that effectively prevents HBV infection [7,8]. Further, in addition to serving as vaccines against the viruses from which they are derived, VLP can function as scaffolds for the presentation of epitopes from any source [9,10,11,12,13]. Most VLP vaccines are formulated with adjuvants and the immunogenicity observed in the preclinical studies need to be confirmed in human trials. Hence, thanks to the high immunogenicity typical of virus particles, VLP serve as platforms for the presentation of a wide variety of potential vaccine epitopes.

### 2.1. VLP-based Technologies

VLP are naturally biocompatible. They are originated from two groups of viruses, non-enveloped and enveloped, and both groups have been used to display foreign antigens [14]. They have no viral genome and are, therefore, not contagious. VLP are effectively eliminated or degraded, which limits the occurrence of side effects [15]. They present epitopes in dense repetitive arrays, making them effective scaffolds that can elicit antibodies to multiple substances (Table 1).

Peptides, small-molecule haptens, and self-antigens can elicit high-titer antibody responses when presented on the surface of a VLP [32,33,34,35]. VLP immunogenicity, however, is not due to multivalence alone. Most VLP are small particles with a diameter in the range of 25 to 100 nanometer (nm), a size that allows optimal entry into lymphatic vessels, passive drainage to the subcapsular region of lymph nodes (LN), and uptake by professional antigen-presenting cells. Studies in mice conditionally depleted of dendritic cells (DCs) confirm that small particles drain freely to the LN [36,37], while DC transport large particles from the injection site to the LN [38].

There are two primary approaches for the presentation of epitopes on VLP. First, synthetic peptides containing the desired target epitope can be cross-linked chemically to the VLP surface. VLP-based vaccines produced in this way are very effective in generating an immune response against display molecules and inducing high titers of neutralizing antibodies [19,20,39,40]. In this approach, the VLP, the epitope, and the linker are synthesized separately and combined later in the cross-linking reaction to produce the vaccine. The chemical cross-linking process increases the cost of production [41] but avoids potential protein folding/self-assembly issues that sometimes occur due to genetic fusion. The chemical approach also sometimes allows for higher display valences. Variations on this cross-linking theme include the linkage of foreign peptides and proteins to VLP using enzymatic methods such as Sortase [42] or the so-called Spycatcher [43] technologies.

In the second approach, foreign peptides can be inserted genetically into the coding sequence of a viral structural protein. When expressed in an appropriate host, the protein self-assembles into a VLP with the peptide exposed on its surface. The most widely used VLP platforms for genetic peptide display are based on the woodchuck hepatitis virus (WHBV) [44] and several RNA bacteriophages [17]. Genetic insertion of foreign sequences sometimes prevents the recombinant protein from folding properly [2]. In the case of HBV core proteins, this problem has been largely overcome through the use of a series of genetic variants that can be employed in a combinatorial fashion to find a VLP that tolerates almost any desired insertion. In the case of RNA phage MS2 VLP, peptides are inserted into a surface loop of coat protein (the so-called AB loop). The wild-type coat protein is nearly always destabilized by such insertions, but a genetically engineered single-chain dimer version tolerates the vast majority of small peptide insertions, making it a nearly universal peptide display platform [22,32,45]. It is a consequence of the increased thermodynamic stability conferred by the covalent joining of two subunits associated non-covalently [46].

Whether displayed by genetic insertion or by chemical conjugation, a peptide linked to a VLP has the ability to elicit a high-titer, long-lived, epitope-specific antibody response. Naturally, each method has its advantages and liabilities. As mentioned above, genetic insertion sometimes fails to yield a VLP as a result of improper recombinant protein folding [2]. Chemical cross-linking of a synthetic peptide to a preformed VLP, on the other hand, obviously avoids this problem. Genetic fusions, however, can yield a more uniform product and can be produced biosynthetically in a single step and often in very high yields. Further, at least in the case of MS2 VLP, the genetic insertion has the added advantage of enabling epitope identification in a process akin to phage display by affinity-selection on antibody targets from complex random-sequence or antigen fragment libraries [47].

**Figure 1:**
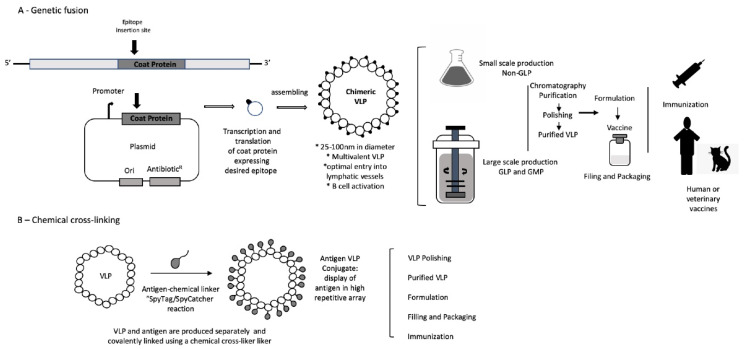
Illustration of virus-like particles (VLPs) production using different approaches. (**A**) Production of chimeric VLP using genetic insertion. The foreign antigen is fused to the coat protein by genetic engineering, and then chimeric VLP is expressed in a suitable host system. (**B**) Chimeric VLP is generated by chemical conjugation of foreign peptides to the surface of the VLP. The VLP production can be carried out on a small scale for scientific research, while under the current GMP, it can be produced on a large scale for human or veterinary.

VLP can be recombinantly expressed in a variety of hosts, including bacteria, yeast, plant, insect, and mammalian cells [48], and is manufactured economically in large amounts with high purity under the current Good Manufacturing Practices (GMP) (Figure 1). Due to their biocompatibility, solubility, efficient uptake, and nanoscale dimensions, VLP also have potential as drug delivery vehicles [49]. The choice of expression host and fermentation process can affect the VLP yield, the integrity, the scale, the cost of production, and the purity of the final product, all factors that can have a significant impact on vaccine stability, efficacy, and safety [22,50,51].

Even though some VLP vaccines have been approved by the Food and Drug Administration (FDA) for human use, every decoration of a VLP with a new epitope creates an unknown particle that must be well characterized. Different versions of a VLP vaccine can be generated based on its size, and the nature or length of the epitope displayed, so the analysis of their molecular nature becomes essential and requires an appropriate method to confirm its structural properties. The most utilized methods for VLP characterization are analytic ultracentrifugation, high-performance chromatography, cryo-electron microscopy, atomic force microscopy, transmission electron microscopy and dynamic light scattering. These methods confirm the integrity of the VLP for epitope display, which is a key factor for inducing a functional immune response against the target antigen [52].

### 2.2. VLP Toxicology

The source of the potential toxicity of the VLP vaccine comes from the immunization or the immune response it induces. Since VLP-based vaccines are potent inducers of the immune response, safety studies are necessary to examine the potential toxicities of its components and pharmacodynamic. Any of a VLP’s main components—the protein capsid (coat protein), any packaged nucleic acids, the chemical cross-linker, and the displayed antigen—could present some potential toxicity. If an adjuvant is used for vaccination, its potential toxicity must also be considered.

As mentioned above, sometimes, VLP from different viruses such as Q-β, Cowpea chlorotic Mottle Virus (CCMV), and MS2, can incorporate nucleic acids during their expression in the host [53,54]. In the case of the MS2 VLP, this event represents as much as 25% of the particle’s mass [45]. Rather than a liability, this RNA component enhances the immune response because of its adjuvant properties as an activator of TLR7 and TLR8 [55,56]. The two prophylactic HPV vaccines approved by the FDA are highly immunogenic and safe [52]. Imiquimod and Resiquimod are ssRNA analogs used as potent anti-viral and anti-tumor therapy with a high degree of safety and no organ toxicity [35].

The succinimidyl (SMPH), an amine-to-sulfhydryl cross-linker used to conjugate the antigen to the VLP chemically, forms an irreversible thioether between the cross-linker and cysteine residue of the antigen. The lability of SMPH in aqueous solution and relatively low concentration (1–2 mM) used in the peptide conjugation process minimizes the possibility of a toxic effect [35]. As an alternative, the use of the bio-orthogonal copper (Cu)-free click chemistry represents an excellent choice for binding conjugated epitopes to VLP. Compared to SMPH, this method has proven to be more effective, safer, and more immunogenic [21,24].

The display of a chemically conjugated antigen can present a potential for toxicity, especially when it is a self-antigen. Unconjugated antigen may bind to healthy cells and produce some adverse effects. The manufacturing process should remove or reduce the concentration of unconjugated antigen to the levels where no biological effect occurs. However, when the removal of self-antigen excess is not possible, its toxicity must be addressed. This problem does not exist with autoantigen genetic conjugates [35].

Adjuvants, such as inorganic salts (alum), oil emulsions (MF5), and lipid A component (MPL), can be used to improve vaccine immunity and may cause adverse reactions. Luckily, VLP vaccines by themselves induce such a robust immune response that the adjuvant may be unnecessary, but if the use of an adjuvant is required for optimal immune response, its potential toxicity must be addressed [57].

The immunization process or the resultant immune response might be a source for local or general systemic toxicities and, therefore, preclinical studies must be representative of the vaccine route and formulation intended for clinical use. The constituents required to modulate the immune response should also be present. When possible, batches of a vaccine developed for clinical vaccination should be used in toxicology study, so the product is close as much as possible to the one that enters practice. Production scale up, for example, can introduce subtle changes that can have significant effects on product quality.

Appropriate animal toxicology assessments can determine the viability of candidate VLP vaccines. Repeated dose toxicity studies in individual animal species are sufficient to validate new prophylactic vaccine products [58,59]. However, for VLP that present autoantigens, non-clinical studies in two species may be required. To characterize the cytotoxic T lymphocyte (CTL) response, the rodent is an appropriate model. Some mouse strains show a differential tendency for a Th1 or Th2 response, a critical consideration in choosing an animal recipient [60], especially for VLP vaccines designed for tumor therapy where the precise nature of the immune response can have vital importance. Rabbits represent a good choice for toxicology studies because they show the required humoral response and are large enough to be used for full-dose administration to human recipients [61].

Toxicology studies are usually performed in several doses. However, single doses may be necessary if the immune response induced by the first administration alters the reaction of the subsequent ones. Such repeated dose studies are critical to support the safety profile of vaccines under development [61]. These studies provide useful information for VLP platforms inducing B or T cell responses, as this application involves more than one dose or immunization. The dose, the route of administration, the system distribution, and the degradation of the VLP must also be considered for toxicology studies. Physiological effects outside the immune system are of concern, especially for vaccines with autoantigens [24,62].

### 2.3. VLP and the Lymphatic System

The draining of nanoparticles to LN is an essential property of VLP vaccines. As mentioned above, most VLP platforms have diameters ranging from 25 to 100 nm, making them small enough to be drained into the lymphatic vessels, whose diameters vary from 10–60 μm up to as much as 2 mm. Ultra-small nanoparticles such as Pluronic-stabilized polypropylene sulfide (PPS) (~25–100 nm) injected intradermally are transported highly efficiently into the lymphatic capillaries and their draining LN [63]. EαRFP, a recombinant protein injected subcutaneously (s.c.), is transported to the LN in 18 h [64]. In one lymphatic trafficking study, VLP of bacteriophage Qβ (30 nm of diameter) labeled with Alexa-488 were injected in C57BL/6 mice. Fluorescent polystyrene nanoparticles with diameters of 20, 500, and 1000 nm were also injected. Within two hours, VLP and 20 nm beads were detected in the subscapular sinus of the popliteal LN, where they were associated with LN-resident DCs, B cells and macrophages. This pattern is compatible with drainage of afferent lymphatic capillaries. By 48 h, they were localized at the subcapsular, paracortex, and cortex areas of the LN in the vicinity with B cell follicles [36]. Larger 500 and 1000 nm beads needed 24 to 48 h to reach the popliteal LN and colocalized to areas where DCs reside. They did not efficiently enter lymphatic vessels and were possibly carried into the LN by specialized cells immigrating from the skin. One week after injection, 20, 500 and 1000 nm particles were still present at the injection sites and popliteal LN. At this time, the presence of VLP was reduced, which may be due to its degradation. The transport differences of these nanoparticles indicate size-dependent entry and distribution into the lymphatic system [36].

By freely entering the afferent lymphatic vessels and the LN subcapsular sinus where they encounter B cells and resident DCs, the VLP can efficiently initiate a humoral immune response. The VLP then spreads through the small gaps (0.1–1 μm) of the sinus floor to the follicular area and interacts with naive B cells [65]. The VLP can be kept for an extended period in the germinal center by follicular DCs, leading to the clonal expansion and the development of a long-lived antibody response. Alternatively, the VLP can be processed by DCs in the paracortex region, where a substantial fraction of the DC population is immature and able to process new antigens [66,67], initiating an immune response and the development of effector mechanisms [37]. DCs are also involved in the transport of large particles from the interstitial space to the lymphatic vessels through an active mechanism involving cell adhesion molecules [38]. Larger particles confined to the interstitial space before entering the LN are susceptible to the action of phagocytic cells [36], reducing their ability to be drained or transported.

VLP represent a reliable antigen delivery system whose route of administration determines the strength of the immune response [37]. Studies of different immune routes using simian human immunodeficiency SHIV VLP (approximately 90 nm in diameter) and near-infrared (NIR) fluorescent dyes in SKH-1 hairless immunocompetent mice show significant differences in particles trafficked into LN. Five minutes after intradermal injection, the VLP can be detected in the inguinal, popliteal, lumber, and sciatic LN, remaining detectable for up to six days [37]. When the VLP reach the subcapsular sinus, a larger population of B cells is activated and migrate from the border between the T cell zone and follicle to receive proliferation signals from antigen-specific T helper cells [68]. The increase in B cell proliferation can lead to increased levels of antibody-secreting plasma cells. Hence, an increase in VLP uptake in the LN can result in a more robust immune response. Further studies using SHIV intradermal (i.d.) immunization in C57Bl/6 mice showed a healthy antibody level production, possibly due to the increase in LN involvement leading to a better overall immune response [37].

Through effective drainage, the VLP can generate antibodies with higher affinity for specific epitopes through somatic hypermutation (SHM) at the germinal center. VLP-stimulation of gene regulation, cytokine production, and antigen-specific antibody production provide important information for the development of VLP-based vaccine [69]. Intradermal vaccination seems to produce a higher activated germinal center B cells [37].

Spleen cells from C57BL/6 mice i.d. immunized with SHIV VLP were analyzed by flow cytometry for the expression of various activation markers. The results demonstrated that most B cells (B220^+^) were also positive for Fas and GL7 proteins, typically expressed on activated germinal center B cells. The population of B cells was also highly positive for the expression of CD80, a co-stimulatory molecule required for T cell activation and survival. When up-regulated on activated B cell, CD80 provides T cell co-stimulation through CD28 signaling. In the same experiment, the B220^+^PD1^+^ population increased after VLP immunization. PD-1 is a member of the CD28/CTLA4 family that is up-regulated on activated macrophages, T cells, and B cells. This increase in the population of activated and germinal center B cells, as evidenced by the expression of specific surface markers, might explain why i.d. vaccination leads to significantly higher affinity levels of antibody production [37]. VLP immunization was also effective in inducing an expansion of CTLs, as demonstrated by a cytotoxicity assay using a simian immunodeficiency virus (SIV) gag peptide pool. CTLs are an essential component of the cellular immune response, where they induce the death of virally infected cells and tumor cells. The i.d. SHIV VLP immunization produced higher numbers of CTLs than other vaccination pathways [37].

Humoral and cell-mediated responses require the activation of multiple effector mechanisms to identify virus infected cells and tumor cells, which may not be achieved by all vaccines. Cancer vaccines rely mostly on the development of antigen-specific antibodies and on the generation of antigen-specific CTLs to recognize distinct antigens present on the cancer cell surface to eliminate them. It is well established that through cross-linking B cell receptors (BCRs), VLP activate the B cells, originating a strong activation response [70]. VLP can be modified to present a variety of epitopes, and to display B cell epitopes in a rigid, organized, and repetitive manner to effectively induce the production of neutralizing antibodies [71].

### 2.4. VLP Vaccination Can Directly Activate B Cells

B cells are an integral part of the adaptive immune response. The identification of the subtype of B cells that binds to VLP can help to understand the mechanisms of B cell activation, differentiation, gene regulation, cytokine, and antigen-specific antibody production. In an explicative study, the SIV, SHIV, and chimeric influenza HA/SIV (SIV gag plus influenza hemagglutinin) VLP, known to induce high titers of antibodies, were incubated with mouse splenocytes [72]. All three types of VLP bound directly to CD19^+^ naive B cells but not to CD3^+^ T cells, as expected by the fact that the mouse CD4 cannot be properly recognized by HIV envelope protein. Dose-dependent binding and unlabeled VLP competition assays showed that the VLP binding to naïve B cells is specific [72]. Indeed, B cells may be specific for VLP antigens, and binding occurs via their antigen receptor (BCR) rather than toll like receptors (TLR), and induces up-regulation of the activation markers CD69 and CD86 [72].

Among the population of naïve B cells (B220^+^IgM^+^), conventional B2 cells (CD43^−^CD5^−^) increased after treatment with VLP, compared with PBS control, while the B1 cell subpopulations B1a (B220^+^IgM^+^CD43^+^CD5^+^) and B1b (B220^+^IgM^+^CD43^+^CD5^−^) did not change after VLP exposure [72]. This data indicates that incubation of naïve B cells with VLP can stimulate B2 cells to expand in vitro.

Therefore, some VLP are capable of directly inducing the activation of the humoral response through specific binding to naïve B2 cells in vitro [72]. However, immunization with other VLPs may produce different results. A study with HPV16 vaccination, for instance, showed the ability of HPV16 VLP to activate naïve B cells, although, in this case, an increase in B1 cell subpopulation was reported [73]. Another study using Qβ and AP205 VLP induced the expansion of B1 cells from the marginal zone of the spleen [74]. The nature of the VLP structure and the type of infection are speculated to be involved in these differences.

**Figure 2:**
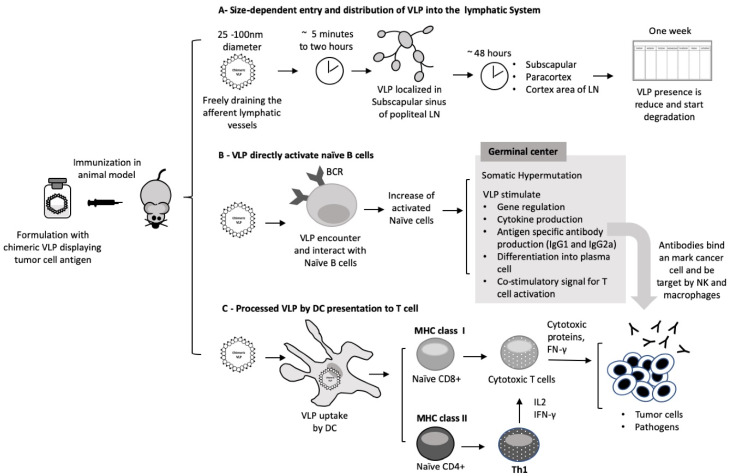
Illustration of virus-like particles (VLP) triggering immune response. (A) The draining of nanoparticles to the lymphatic system is an essential property of nanoparticles. (B) VLP can directly activate naïve B cells and produce a long-lasting immune response. (C) VLPs processed by DC cells trigger immune response and development of effector mechanisms.

VLP purification after expression in *Escherichia Coli* does not remove all the bacterial endotoxin, leaving traces of lipopolysaccharide (LPS) in the VLP formulation. Are the VLP alone responsible for inducing the expansion of naïve B cells, or is the residual endotoxin involved? Spleen cells incubated with VLP, LPS, or anti-CD40 antibody in the presence or absence of polymyxin B (PMB—an antibiotic that blocks LPS activity) helped to answer this question. The naïve B cell proliferation was reduced in the presence of LPS and PMB but was not affected when treated with VLP and anti-CD40 in the presence or absence of PMB, showing that activation of naïve B2 cells by VLP is not dependent upon the presence of endotoxin [72]. The same study showed that in the supernatant of naïve mouse splenocytes stimulated by treatment with VLP, the expression of IL-12, MIP-1α, and MIP-1β is elevated, while the expression of IL-4 and MCP-1, which favor IgG1 antibody production, was decreased. Therefore, VLP stimulation is conducive to IgG2a class-switch recombination (Figure 2) [72].

B cells can respond to antigen in a T-dependent or T-independent way. In both cases, besides antigen binding through the BCR, additional signals are required to induce B cells to proliferate and differentiate into plasma cells producing antibodies [75]. VLP bind and activate naive B cells, but can VLP induce B cells to differentiate into plasma cells? Splenocytes incubated for 48 h with VLP were transferred to a SIV VLP-coated polyvinylidene fluoride filter plate for 3 h at 37 °C. The ELISPOT assay showed that VLP treatment induces the differentiation of activated B cells into plasma cells, at least in vitro. These data were confirmed by real-time PCR analysis where the levels of Blimp-1 and XBP-1 increased after splenocytes incubation with VLP; these two proteins are essential for the differentiation of plasma cells. The level of antibodies produced after plasma cell differentiation was evaluated by ELISA, with a remarkable increase in both IgM and IgG2a, confirming that VLP stimulated a humoral response in vitro [72]. VLP immunization can also stimulate B cell differentiation into a plasma cell and class-switch recombination in vivo [72].

### 2.5. VLP Can Activate the Complement System

Proteins on the surface of VLP, like those of the viruses from which they are derived or other pathogens, are very organized and repetitive. Hence, an active binding to natural IgM antibodies or IgG, can recruit complement component 1q (C1q) and activate the complement cascade. In addition, protein C and other pentraxins can bind to the surface of VLP, also activating the classical complement cascade, and facilitating their uptake by DCs and macrophages. After being taken up by these antigen-presenting cells (APCs), the VLP reaches the endosome-lysosome compartment and is degraded into peptides. These peptides through MHC class II molecules are carried to the cell surface and presented to CD4^+^ T helper cells. The vaccine antigen can alternatively be presented by MHC class I molecules to induce CD8^+^ T cell responses, an essential requirement for therapeutic vaccine’s candidates [76].

### 2.6. VLP Vaccination Strategy, Regimen, and Dose 

Vaccination has the primary purpose of producing long-lasting protection against diseases. The choice of appropriate vaccine strategy, regimen, and dose is crucial for the success of vaccination. It becomes especially concerning when immaturity or senescence of the immune system can affect the efficacy of the immunization [77]. Different strategies of prime-boost vaccination against infectious diseases searching to improve humoral and cellular immunity have been studied [78,79]. These heterologous strategies induce efficient humoral and cellular responses to the same antigen presented by two different delivery systems. Priming with a DNA vaccine or viral vector followed by boosting with a protein-based vaccine usually induces a strong cellular immune response, with higher and more specific antibody production as compared to homologous delivery systems [80]. In the circumstances where homologous protein-based vaccination induces strong humoral response but weak cellular immunity, the heterologous approach can be more productive. Below are examples of the prime-boost regimen:

- DNA prime/viral vector boost, viral vector prime/DNA boost;
- DNA prime/protein boost, DNA prime/peptide boost;
- Protein prime/viral boost, viral prime/protein boost;
- DNA prime/VLP boost, VLP prime/live vector boost.

They combine a better antibody and CD4^+^ T cell response induced by protein antigen, efficient stimulation of T cell response by DNA vaccines, and improvement of the CD4^+^/CD8^+^ T cells and antibody response by recombinant viral vectors. Similarly, different vectors have different immune characteristics and, therefore, induce a unique immune response to the immunodominant epitope, and reduce the immunity against the vector [79].

The choice of the optimal time and the frequency of repeated boosts can impact the quality of the immune response [81]. Understanding the process of establishing immune memory is valuable information for choosing the interval between the primary immunization and booster injections. Memory T cells with high proliferative potential are formed several weeks after the prime immunization, suggesting that the boosting should occur at least two or three months after the first immunization. A similar concept applies to antibody responses, as memory B cells need to undergo a germinal center response to develop [82]. Repeated boosting drives T cell towards terminal differentiation and recruits a subset of a previously generated memory cell; as a result, a heterologous population of memory T cells at several different stages can be found [78]. An appropriate immunization schedule can take advantage of this process.

The establishment of permanent protective T cell memory may depend on the antigen load and its kinetics. The possibility of mimicking pathogen replication enhances the T cell activity. A study using choriomeningitis virus (LCMV) in mice with different doses that prolong the antigen exposure showed a significant effect on the immune response, where high doses of the virus led T cell exhaustion, while low doses induced potent and durable T cell activation [55]. The ideal vaccination schedule starts with low doses, followed by a peak dose and finishes with low doses, instead of repeated doses of equivalent vaccine concentration [83]. This approach appears to be more suitable for therapeutic vaccines. In contrast, for prophylactic vaccines, when an effective B cell response is wanted, a simple vaccination schedule with an injection in a month or more apart may be preferable. The immune system can generate a reservoir of antigens in the germinal center, keeping B cells stimulated, which results in a stronger antibody response. Memory B cells and plasma cells favor this event that may contribute to the success of inactivated virus vaccines [76].

### 2.7. Usage of Adjuvants 

Adjuvants may be needed to increase the antigen reservoir and enhance vaccine efficacy. For almost a century, Aluminum and Freund’s adjuvants were used to improve vaccine immunogenicity. Nowadays, many others have been developed [84]. Preclinical studies show that some VLP vaccines do not need additional adjuvants to produce a robust immune response since they have intrinsic adjuvant properties to activate TLR. In mice, the ssRNA into the VLP [85] induces higher antibody titers of IgG2a, IgG2b and IgA isotypes [86], while VLP with the ssRNA removed, elicit IgG1 antibodies, indicative of a Th2 response [18,87]. Overall, VLP are very immunogenic, even in the absence of TLR-mediated stimuli. However, TLR ligands increase the immune response of a vaccine, as observed in Cervarix, an HPV-derived VLP used in combination with MPL, which is a ligand for TLR4 [40]. The TLR9 ligands CpG-containing oligodeoxynucleotides (CpG-ODNs), are also used in vaccines to enhance B and T cell responses to a given recombinant epitope. CpG package into VLP is resistant to DNase I digestion and does not induce the undesirable side effect (splenomegaly and lethal toxic shock) caused by the free form of CpG. Vaccination with CpG-loaded VLP protected from infection with vaccinia virus and eradicated established solid fibrosarcoma tumors [84]. Therefore, VLP presenting the CpG might represent a valuable approach to induce CTL responses [76]. Another way to enhance the VLP cell-mediated immune response is to present the universal T-assisted pan-HLA-DR-binding epitope (PADRE) on the nanoparticles. The RHDV VLP carrying PADRE and displaying HPV16 E6 peptides successfully prolonged survival of mice with pre-existing HPV tumors [25].

## 3. VLP as Cancer Vaccines

In the past decade, cancer immunotherapy with checkpoint inhibitors has achieved a remarkable clinical outcome in patients with advanced stages of cancer [88]. However, the high cost for such therapies and the low percentage of patient responders highlight the need to find new immune-based strategies to fight cancer, which are both cost-effective and efficacious for a broad patient base [89,90]. Human cancer emerges through a combination of genetic and epigenetic changes that favor its survival. The tumor-associated antigen and tumor-specific antigens (neo-antigens) also create an opportunity for tumor detection and destruction by the immune system. However, cancer cells have adopted several strategies to evade immune recognition, leading to a state of tumor tolerance [91]. Biological response modifiers (such as vaccines) have the potential to fight cancer and can be used to break tumor tolerance. The vaccine must deliver a large number of selected cancer antigens to promote B cell and DC activation and antigen presentation to T cells. As mentioned above, with the VLP platform, even short peptides identified in tumor cells (that otherwise would be quickly cleared) can be presented to DCs to induce cellular or humoral response against cancer. Favorable results obtained by preventive VLP-based vaccines against cancer caused by infectious diseases—for instance, HBV and HPV—reinforce this concept, and many attempts are now ongoing for the development of VLP-based vaccines against many non-infection-related cancers. In the following sections, we review some relevant examples.

### 3.1. Cervical Cancer 

The current FDA-approved HPV vaccines are derived from HPV capsid protein L1 formulated with aluminum-containing adjuvants—aluminum hydroxyphosphate sulfate for Gardasil and the AS04 adjuvant system, composed of aluminum hydroxide and the TLR ligand 3-*O*-desacyl-4.-monophosphoryl lipid A (MPL) for Cervarix [92]. These vaccines are highly immunogenic and induce long-acting neutralizing antibodies. Their formulation is complex and expensive, which prohibits affordability in developing countries, where 85% of cervical cancer cases occur.

A preclinical study showed that another VLP-HPV vaccine using a neutralizing epitope from the HPV minor protein L2 could offer a robust immune response using a lower-cost production methodology and improved clinical applicability [23]. For this new approach, HPV16L2 was displayed at the N-terminus of MS2 VLP and at the AB loop of PP7 VLP. The L2-MS2 and L2-PP7 VLP were produced in a dry powder version using Spray Dryer (SD) and had their thermostability storage testing at 4 °C, 37 °C, and room temperature for one month. Spray Transmission Electron Microscopy (TEM) showed that SD and storage at the tested temperatures do not affect the nanoparticles’ integrity or stability, preserving their ability to generate HPV-neutralizing antibodies. Immunization with reconstituted L2-MS2 VLP elicited high titers of anti-L2 IgG. The immunized mice were challenged with HPV Pseudo Virus (PsV), and their protection was compared with that induced by Gardasil immunization. When tested with two homologous PsV types (PsV31 and PsV45), L2-MS2-immunized mice were efficiently protected from both infections, while Gardasil failed to protect against the PsVs45 challenge. This work showed that L2-MS2 vaccines could be produced in a dry powder version with stability up to seven months at room temperature [23]. For other VLP-based vaccines against HPV cervical cancer, see [62].

### 3.2. Hepatocellular Carcinoma 

The acute and chronic hepatitis caused by HBV can progress to cirrhosis and eventually to hepatocellular carcinoma (HCC). The current HBV vaccines are based on VLP of the S antigen; the most recently approved, HEPLISAV-B, has CpG as adjuvant [92]. These vaccines reduce the occurrence of HBV infection but are ineffective in treating existing HCC. The use of multiple epitopes’ VLP was tested as a vaccine for HCC treatment in a preclinical study. The HBV X protein is highly expressed in HCC. High frequency of epitopes of HBV X displayed at HBV core protein elicited epitope-specific CD8+ T cells and a more forceful response than a single peptide showing that multiepitope-loaded VLP augment immunogenicity for each epitope by increasing the number of CTLs resulting in a significant anti-tumor response [28]. It is a proof of concept of the potential efficacy of VLP-based vaccines for HCC therapy.

### 3.3. Skin Cancer 

Melanoma is an antigenic and immunogenic cancer with well-characterized tumor-associated antigens. Ideally, a melanoma-preventing vaccine should expand the number of spontaneous CTLs produced against the tumor at the tumor site and throughout the body as long-term immune surveillance [93].

A notable example of the potentiality of VLP against melanoma comes from a preclinical study using VLP not decorated with tumor antigens but with inherent immunogenic properties.

In situ vaccination, using the VLP generated by the plant virus Cowpea Mosaic Virus (CPMV), was shown to reduce B16F10 melanoma and generate systemic anti-tumor immunity [29]. The approach of in situ vaccination using VLP is limited, but local immunization can strongly modulate the local microenvironment and the anti-tumor immunity. Empty CPMV (eCPMV) VLP lacking any nucleic acid were tested in in vitro cultures of bone marrow-derived DCs (BMDCs) and primary macrophages from C57BL/6 mice. Twenty-four hours later, an increase in pro-inflammatory cytokines (IL-6, IL-1β, IL-12p40, IFN-γ and TNK-α) was observed, providing evidence that the empty VLP is intrinsically immunostimulatory. Notably, the VLP were produced in plants and, therefore, were endotoxin free. When tested in non-tumor-bearing mice, inhalation of eCPMV activated Ly6G+ neutrophils that up-regulated the CD11b activation marker and the CD86 co-stimulatory molecule. Inhalation of eCMVP by mice bearing lung melanoma metastases, induced by an intravenous (i.v.) injection of B16F10 cells, alters the lung microenvironment, with a significant increase in tumor-infiltrating neutrophils (TINs) and CD11b^+^Ly6G^+^-activated neutrophils as well as a reduction in CD11b^−^Ly6G^+^ quiescent neutrophils. In addition, weekly intratracheal administration of 100 µg of eCPMV reduced the number of metastatic foci both in mice i.v. injected with B16F10 cells and in mice developing spontaneous lung metastases after a subcutaneous challenge with 4T1 mammary cancer cells [29]. Mice depleted of neutrophils and those lacking IL-12, IFN-γ or T, B, and NK cells failed to respond to the eCPMV inhalation therapy, confirming that neutrophils can disturb the tolerogenic nature of the tumor microenvironment and orchestrate an immune response that leads to tumor elimination. It probably occurs due to activation of pre-existing or de novo-induced anti-tumor responses, or both [29]. Administration of eCPMV in situ was also effective for treating dermal tumors. When the VLP were injected intra-tumorally in mice bearing cutaneous tumors induced by B16F10 melanoma cells or CT26 colon cancer cells, tumor growth was delayed. In the case of B16F10 melanomas, 50% of the treated mice rejected the tumor. These surviving mice were also protected from a re-injection of B16F10 cells, indicating the induction of a protective systemic immune response.

Another important preclinical study was based on the use of VLP decorated with the H-2D^b^ immunodominant epitope of the human glycoprotein 100 (gp10025-33) [26], a melanoma-associated antigen [94]. The epitope was incorporated into rabbit hemorrhagic disease virus (RHDV) VLP in one, two, or three copies, resulting in gp100.1L, gp100.2L, and gp100.3L VLP, respectively. All three VLP induced a robust CD8^+^ T cell proliferation, and gp100.2L and gp100.3L also induced a significant enhancement of IFN-γ production [26]. The activation of T cell proliferation was confirmed in in vivo experiments for gp100.2L and gp100.3L. These two versions of VLP also showed a therapeutic effect against murine melanomas. Mice vaccinated with the VLP remained tumor free over 60 days, which is an indication of the induction of a specific anti-tumor immunity. The mono or dimannosylation of gp100-VLP enhanced VLP uptake into APCs, but the anti-tumor T cell response was only enhanced by dimannosylation [26].

Another proof of the ability of VLP-based vaccines to prevent B16F10 melanoma comes from a study in which a recombinant cucumber-mosaic VLP (CuMV) (~ 30 nm) was used. [24]. The universal tetanus toxoid (TT) epitope TT830-843 has been genetically fused to CuMV (CuMV_TT_) and is displayed in the interior surface to avoid interference with TT-specific antibodies. The TT epitope serves as an additional T helper cell epitope, especially in elderly patients. CuMV_TT_ was labeled with AF488 and was used in the study of drainage dynamics. The study showed that fast and effective drainage of VLP, that reached the regional LN in just one minute. A version of these VLP expressing the H-2D^b^ restricted p33 peptide from LCMV antigen (CuMV_TT_p33-VLP) was formulated with the micron-sized microcrystalline tyrosine (MCT) adjuvant. MCT facilitated depot formation and prolonged the release of the VLP up to 9 days compared to 4 days of the free VLP form. With only one vaccination, CuMV_TT_p33 VLP formulated with MCT can induce high levels of p33-specific T cells and release of the cytokines IFN-γ and TNF-α. When this formulation was tested in mice bearing tumors from B16F10 melanoma cells expressing the p33 antigen, tumor progression was inhibited with a significant increase in total CD8^+^ T cells and p33-specific T cell infiltration, showing proper anti-tumor protection [24].

A recent study proposed a therapeutic vaccine for melanoma, in which the loaded CpG Qβ-VLP and the desired epitope are combined with bio-orthogonal copper-free click chemistry; Dibenzocyclooctyne NHS ester (DBCO) is used as a cross-linking agent. This new method for peptide conjugation was shown to be superior to SMPH not only for coupling efficiency but also for inciting CTL and IFN-γ response in vivo. The developed vaccine was created by the conjugation of peptides from tumor-specific germline (immunopeptidomics) and mutated (whole-exome sequencing) CTL epitopes from B16F10 melanoma cells. Three different multitarget (MTV) vaccines were originated: 1) germline-multitarget vaccine (GL-MTV), 2) mutated epitope vaccine (mutated-MTV), and 3) a combination of both (Mix-MTV). Subsequently, they were tested in mice with transplanted B16F10 melanoma tumors in combination with anti-CD25. It was observed that the three vaccines inhibited the tumor progression, the Mix-MTV vaccination being more effective for anti-tumor protection. It was also the only one that showed significant tumor growth reduction. Compared with the control group and mutant MTV, the Mix-MTV vaccine was more effective in increasing CD8^+^ T cell density. For Mix-MTV vaccination, higher IFN-γ production was also observed. Data show that the combination of germline and mutant epitopes in a vaccine is more effective against growing B16F10 melanoma tumors [21].

### 3.4. Pancreatic Cancer 

Pancreatic cancer is resistant to many forms of treatment, highlighting the urgent need to develop novel therapies to fight this neoplastic disease.

Mesothelin (MSLN), a glycoprotein membrane whose expression is limited to mesothelial cells, is overexpressed in most pancreatic cancers and is involved in tumor adhesion and dissemination, making it a robust therapeutic target [95,96]. By presenting the human (h) MSLN on simian-human immunodeficiency VLP (SHIV), a pancreatic VLP-hMSLN vaccine was created [30]. The efficacy of VLP-hMSLN was tested in Panc02 cells implanted orthotopically in mice, showing significantly lower tumor progression and reduction in tumor mass and the survival of 60% of vaccinated mice. Protection was due to the induction of high concentrations of specific anti-hMSLN antibodies and the release of IFN-γ, accompanied by a strong and specific CTL response and a reduction in regulatory T cells (Treg; CD4^+^FOXP3^+^ICOS^−^) [30]. The hMSLN was a xenogenic antigen in mice, even if human and mouse MSLN has 60% homology. To verify whether the mouse (m)MSLN can break self-tolerance, mMSLN was displayed in the SHIV VLP (VLP-mMSLN). Immunization with this murine VLP version efficiently induced CD8^+^ T cell activation, reduced tumor volume, and survival of tumor-bearing mice [30].

Another pancreatic vaccine candidate was previously developed using SIV expressing mTrop2 (mTrop2 VLP) [31]. Trop2 is a surface glycoprotein overexpressed in pancreatic cancer and is associated with poor patient survival [97]. The efficacy of mTrop2 VLP vaccination was tested in the syngeneic pancreatic cancer murine model. The immunization was able to break tolerance and generate a cellular and humoral response overcoming some of the suppressive tumor microenvironment agents. The results showed a reduction in tumor growth, activation and infiltration of CD4^+^, CD8^+^ T cells, and NK cells. VLP vaccination, in combination with gemcitabine, increased the survival of tumor-bearing mice, suggesting that mTrop2 VLP might be the right approach for pancreatic cancer treatment [31].

### 3.5. Colorectal Cancer 

Colorectal cancer (CRC) is distinguished by a high inter-patient and intra-tumor heterogeneity that makes the identification of possible target antigens challenging. Hence, targeting a combination of CRC antigens seems to be a more promising approach. A preclinical study developed a VLP from the VP60 capsid proteins of RHDV displaying at the N-terminus peptide of the murine topoisomerase IIα (T.VP60), survivin (S.VP60) or both peptides (TS.VP60) [27]. These two antigens are involved in relevant mechanisms for tumor growth in both murine and human CRC. CRC cell line MC38-OVA was injected subcutaneously on day 0, and vaccination was performed over three consecutive days (days 7, 8, and 9) and monitored for 100 days. Synthetic CpGs (25 μg) were used as an adjuvant. T.VP60, S.VP60, and TS.VP60 vaccination delayed tumor growth; the first two treatments (T.VP60, S.VP60) improved survival by 60%, while TS.VP60 enhanced it by 73%. All mice that survived were tumor free at day 100; these mice received a second challenge of MC38-OVA, and none of the mice developed the tumor with 100% of survival up to the completion of the 200 day trial. The results suggest the development of a systemic memory response and demonstrate that a VLP vaccine with multiple epitopes can be an effective treatment strategy for CRC [27].

Other CRC-associated antigens are under investigation as possible targets of VLP-based vaccination. The Cluster of Differentiation 44 (CD44) is an adhesion molecule in the extracellular matrix that induces tumor cell invasion and metastasis. The variant form CD44v6 is a CRC cell marker and its positiveness is correlated with poor survival in CRC patients. CD44 variants stabilize cystine/glutamate antiporter protein xCT (see below) on the cell surface of several human carcinomas. The presence of xCT itself is an independent predictor of recurrence in CRC patients and is correlated with lymphatic and venous invasion. xCT is associated with CRC cancer cell growth, invasion and metastasis, making xCT a reliable target for the development of a CRC therapeutic vaccine [98]. Recently developed VLP-based vaccines targeting xCT [22,99] would be an ideal candidate for preclinical testing in CRC models.

### 3.6. Breast Cancer

Breast cancer is a heterogeneous disease. Triple-negative breast cancer (TNBC) is an invasive form of breast cancer, with high propensity to relapse, and characterized by the absence of epidermal growth factor receptor 2 (HER2), progesterone, estrogen. The presence of cancer stem cells (CSCs) is related to the aggressiveness of TNBC. These CSCs are resistant to conventional therapies and are conducive to tumor development and progression [100,101]. Currently, there is no successful clinical therapy for this subtype of breast cancer. TNBC resists current cytotoxic therapies due to its unique detoxification mechanism, which is greatly promoted by the overexpression of the cystine-glutamic acid transporter xCT (SLC7A11). The xCT protein is overexpressed in several human tumors but not in healthy mammary gland tissue, suggesting that the protein up-regulation occurs only upon oncogenic transformation, contributing to a decrease in patient survival [102]. xCT exports glutamate at a ratio of 1:1 in the exchange of extracellular cystine [103]. Intracellularly, cystine is reduced to cysteine, a limiting precursor in the biosynthesis of glutathione (GSH), which plays an essential role in cellular defense against oxidative stress, reducing diverse reactive oxygen species (ROS) [104]. xCT increases the intracellular concentration of GSH, reduces p38/mitogen-activated Protein Kinase activation, inhibiting ROS and preventing cell apoptosis. As a result, xCT protects CSCs from ROS, making them more resistant to conventional therapies, and promotes tumor growth [105].

Sulfasalazine (SASP) is an FDA-approved drug used to treat chronic inflammatory diseases that inhibits xCT protein function [106]; however, SASP has numerous side effects and, as a result, is not a viable therapeutic option. A vaccine using a genetic fusion of the extracellular domain 6 (ECD6) from xCT protein on the AB loop of bacteriophage MS2-VLP, named AX09-0M6, was used as a prophylactic and therapeutic vaccine in a preclinical TNBC model. The adopted vaccination regimen was able to induce high levels of IgG2a antibodies that bound to mouse and human xCT breast CSCs. Anti-AX09-0M6 antibodies reduced the number and dimension of spheres and increased ROS concentration in all breast cancer cell lines tested, demonstrating that the vaccination with AX09-0M6 prevents BCSC self-renewal. The in vivo experiments showed a significant reduction in 4T1 tumor growth and the number of spontaneous pulmonary metastases, increased natural killer (NK) cell infiltration, and CD8+ T cells, which is indicative of positive tumor microenvironment changes and antibody-dependent cellular toxicity (ADCC). No adjuvants were used in this vaccination approach, and no toxicity was detected [22,99]. Similar results were obtained with an MS2-VLP expressing the ECD3 of xCT, named AX09-0M3 (Rolih et al., data in publication).

The epidermal growth factor receptor-2 (HER2) protein is overexpressed in another aggressive form of breast cancer in humans. Nearly 30% of all invasive breast cancers overexpress HER2. Like many other tumors, HER2+ breast cancer also expresses xCT, and the AX09 VLP against xCT also demonstrated a significant activity against this type of cancer [22,99]. A VLP-based vaccine approach for HER2+ breast cancer vaccination uses Acinetobacter phage AP205 to covalently display the HER2 protein (HER2-VLP) [16]. This vaccine is able to break B cell tolerance and induces a high level of anti-HER2-neutralizing antibodies. Tumor growth was inhibited after HER2-VLP immunization in FVB mice injected with HER2+ transplantable breast cancer cells or HER2+ tumor fragments. In addition, HER2 transgenic mice that spontaneously develop aggressive HER2 mammary carcinomas were tumor free until one year of age after HER2-VLP vaccination, with high levels of anti-HER2 antibodies lasting for up to six months after vaccination [16]. The generated anti-HER2 antibodies bind to mouse and human HER2 in the same way as commercial anti-HER2 monoclonal antibodies.

Although the level of endotoxin was not shown for the AX09 VLP vaccine and not removed from the HER2-VLP vaccine, these vaccines represent an excellent example of how versatile and efficient VLP can be in overcoming B cell tolerance to tumor-associated self-antigens.

## 4. Conclusions

Recent advances in cancer treatment have confirmed that immunotherapy is useful in the ever-evolving fight against cancer. Preclinical studies such as the ones listed above clearly illustrate that VLP can efficiently stimulate the immune system not only against the antigens of the microbial world but also against tumor-associated self-antigens. VLP can target solid tumors or CSCs with the potential to be used as prophylactic or therapeutic cancer vaccines, alone or in combination with chemotherapy, checkpoint inhibitors, or future therapies.

## Figures and Tables

**Table 1:** Summary of the virus-like particle (VLP) vaccines described.

Platforms	Targets	Antigens	Types of Vaccines	References
Bacteriophage AP205	Influenza	M2	Preventive	[9]
	Breast cancer	HER2 protein	Preventive	[16]
PP7	Cervical cancer	L2 epitope	Preventive	[17]
	Cervical cancer	L2 (epitope 17–31)	Preventive	[18]
	HCG	C-terminus	Preventive	[11]
Qβ	Nicotine abuse	Nicotine	Therapeutic	[19]
	Cholesterol	huPCSK9	Therapeutic	[10]
	Alzheimer	pT181	Therapeutic	[6]
	Osteoporosis	TRANCE/RANKL	Preventive	[3]
	Chronic pain	aa 19–241NGF	Therapeutic	[4]
	Diabetes type 2	IL-1β	Therapeutic	[5]
	Diabetes type 2	h IL-1β	Therapeutic	[20]
	Melanoma	GL/mutated-MTV Mix-MTV	Therapeutic	[21]
MS2	Breast cancer	xCT	Therapeutic	[22]
	Cervical cancer	L2 (epitope17-31)	Preventive	[23]
CuMV	Melanoma	TT830–843 epitope	Therapeutic	[24]
RHDV	HPV16 tumor	MHC I-restricted (aa 48–57) HPV16 E6	Therapeutic	[25]
	Melanoma	H-2Db	Therapeutic	[26]
	Colorectal cancer	Topoisomerase IIα, survivin	Preventive	[27]
HBV	Hepatocellular Cancer	HBV X protein-derived epitopes	Preventive	[28]
eCPMV	Melanoma	empty	Therapeutic	[29]
SHIV	Pancreatic cancer	hMSLN	Therapeutic	[30]
SIV	Pancreatic cancer	mTrop2	Therapeutic	[31]
